# HBSP improves kidney ischemia-reperfusion injury and promotes repair in properdin deficient mice *via* enhancing phagocytosis of tubular epithelial cells

**DOI:** 10.3389/fimmu.2023.1183768

**Published:** 2023-05-03

**Authors:** Yuanyuan Wu, Lili Huang, Wenli Sai, Fei Chen, Yu Liu, Cheng Han, Joanna M. Barker, Zinah D. Zwaini, Mark P. Lowe, Nigel J. Brunskill, Bin Yang

**Affiliations:** ^1^ Department of Pathology, Medical School of Nantong University, Nantong, China; ^2^ Department of Cardiovascular Sciences, College of Life Sciences, University of Leicester, University Hospitals of Leicester NHS Trust, Leicester, United Kingdom; ^3^ Nantong-Leicester Joint Institute of Kidney Science, Nephrology, Affiliated Hospital of Nantong University, Nantong, China; ^4^ Research Center of Clinical Medicine, Affiliated Hospital of Nantong University, Nantong, China; ^5^ School of Chemistry, University of Leicester, Leicester, United Kingdom; ^6^ Department of Respiratory Sciences, College of Life Sciences, University of Leicester, Leicester, United Kingdom

**Keywords:** HBSP, innate repair receptor, ischaemia-reperfusion injury, phagocytosis, properdin, repair, tubular epithelial cells

## Abstract

Phagocytosis plays vital roles in injury and repair, while its regulation by properdin and innate repair receptor, a heterodimer receptor of erythropoietin receptor (EPOR)/β common receptor (βcR), in renal ischaemia-reperfusion (IR) remains unclear. Properdin, a pattern recognition molecule, facilitates phagocytosis by opsonizing damaged cells. Our previous study showed that the phagocytic function of tubular epithelial cells isolated from properdin knockout (*P^KO^*) mouse kidneys was compromised, with upregulated EPOR in IR kidneys that was further raised by *P^KO^* at repair phase. Here, helix B surface peptide (HBSP), derived from EPO only recognizing EPOR/βcR, ameliorated IR-induced functional and structural damage in both *P^KO^* and wild-type (WT) mice. In particular, HBSP treatment led to less cell apoptosis and F4/80+ macrophage infiltration in the interstitium of *P^KO^* IR kidneys compared to the WT control. In addition, the expression of EPOR/βcR was increased by IR in WT kidneys, and furthered increased in IR *P^KO^* kidneys, but greatly reduced by HBSP in the IR kidneys of *P^KO^* mice. HBSP also increased PCNA expression in IR kidneys of both genotypes. Moreover, iridium-labelled HBSP (HBSP-Ir) was localized mainly in the tubular epithelia after 17-h renal IR in WT mice. HBSP-Ir also anchored to mouse kidney epithelial (TCMK-1) cells treated by H_2_O_2_. Both EPOR and EPOR/βcR were significantly increased by H_2_O_2_ treatment, while further increased EPOR was showed in cells transfected with small interfering RNA (siRNA) targeting properdin, but a lower level of EPOR was seen in EPOR siRNA and HBSP-treated cells. The number of early apoptotic cells was increased by EPOR siRNA in H_2_O_2_-treated TCMK-1, but markedly reversed by HBSP. The phagocytic function of TCMK-1 cells assessed by uptake fluorescence-labelled *E.coli* was enhanced by HBSP dose-dependently. Our data demonstrate for the first time that HBSP improves the phagocytic function of tubular epithelial cells and kidney repair post IR injury, *via* upregulated EPOR/βcR triggered by both IR and properdin deficiency.

## Introduction

Acute kidney injury (AKI), characterized by a sudden decline of kidney filtration function, is a common health problem associated with high mortality and chronic transformation (1, 2). Ischemia/reperfusion (IR)-related injury is one of important causes of AKI in clinical settings (3). However, due to limited understanding of its underlying mechanism modulating AKI progression, effective and timely intervention is currently unavailable (4).

Erythropoietin (EPO) receptors include the homodimer (EPOR)_2_ initiating erythropoiesis and the heterodimer EPOR/βcR that delivers tissue protection only, thus also known as the innate repair receptor, without role in erythropoiesis (5). The function of EPOR/βcR was mainly discovered *via* its specific ligand EPO-derived helix B surface peptide (HBSP) (6). HBSP attenuated cell death, inflammation and prevented progression of chronic fibrosis after kidney IR injury through multiple signaling pathways, including caspase 9/3, HSP70 and PI3K/Akt/FoxO3a signaling (7–9). It was also reported that EPOR maintains tissue homeostasis (10) by inhibiting the pro-inflammatory functions of macrophages, whilst enhancing their phagocytic functions through activating JAK2/ERK/PPARγ (11). We previously reported that EPOR expression was upregulated by IR at the repair phase of 72 h post injury in mice and further elevated by properdin knockout (*P^KO^*) that also led to more severe damage than wild type (WT) controls (12).

Phagocytosis is a critical process to limit injury and initiate repair after renal IR *via* clearance of damaged cells and inflammation (13). The recognition and uptake of dead cells by a phagocyte usually relies on ‘find-me signals’, opsonization and ‘eat-me signals’ through phagocytic receptors (14). Recently, the complement regulator properdin was found to function as a pattern recognition molecule (PRM) aside from being the sole positive regulator of the alternative pathway activation. Kemper and colleagues reported that neutrophil-released but not serum-derived properdin recognized and bound to apoptotic T cells and facilitated their uptake by macrophages (15). It was also found that properdin binds to the glycosaminoglycan chains of cell surface proteoglycans in apoptotic T cells and is recognized by phagocytic macrophages through heparin receptors. In addition, properdin can also bind to carbon nanotubes and enhance their uptake by macrophages, acting as a PRM and participating in the process of opsonization during phagocytosis (16). Usually produced by inflammatory cells (17, 18), properdin was also expressed on renal tubular epithelial cells (TECs) (12, 19). We revealed *in vitro* that viable TECs produced properdin anchored on damaged TECs subjected to IR-related injury, facilitated the phagocytic clearance of damaged cells and also were directly involved in the phagocytic function of TECs, as semi-professional phagocytes (12).

Investigations whether raised EPOR could function as a compensatory mechanism of phagocytosis in *P^KO^* condition and thus would contribute to the clearance of damaged cells and inflammation after IR injury warranted as proof of principle. Here, based on our previous 72-h renal IR injury model using both *P^KO^* and WT mice, the effect of HBSP treatment was further studied in mice of both genotypes at the same time point. Furthermore, the mechanism of HBSP protection in properdin deficiency mice was explored in particular with focuses on regulating EPO receptors and phagocytosis in TECs.

## Materials and methods

### Mouse kidney IR model

**Figure 1:**
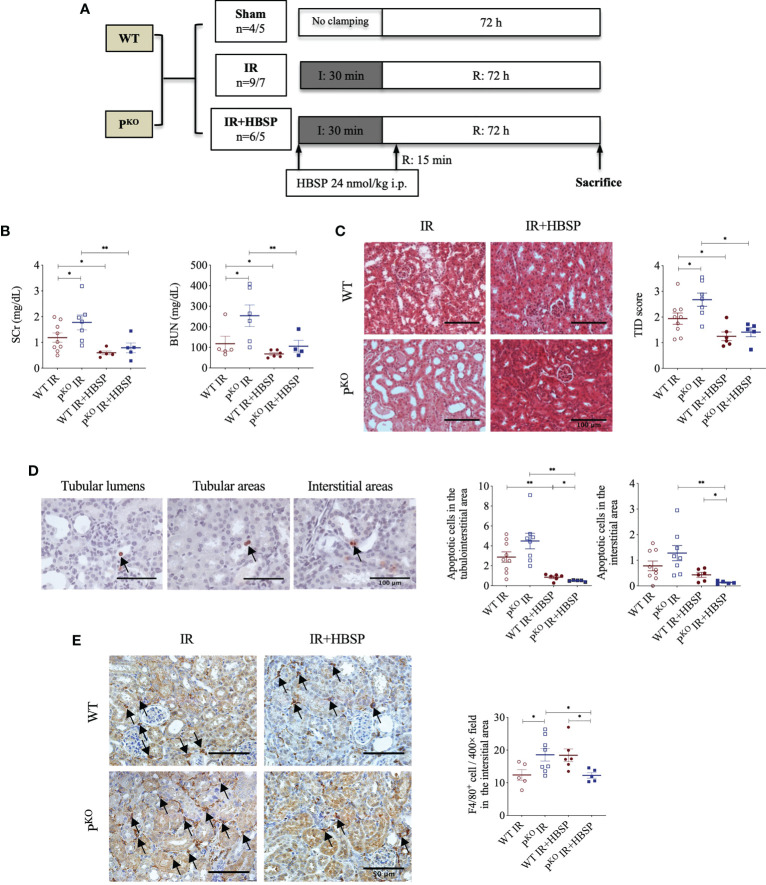
At 72 h, HBSP effectively reduced kidney IR damage in both WT and *P^KO^* mice. **(A)** Animal study design. WT: wildtype; *P^KO^*: properdin knockout; I: ischemia; R: reperfusion; IR: ischemia/reperfusion. **(B)** HBSP significantly decreased the SCr and BUN levels in both WT and *P^KO^* IR mice. **(C)** Representative images of renal cortex with H&E staining shows that HBSP greatly reduced the tubulointerstitial damage (TID) score of the renal cortex in both genotypes. Scale bar, 100 μm; magnification, 20x. **(D)** Representative images of TUNEL staining shows apoptotic cells in tubular lumina, tubular areas and interstitial areas. Scale bar, 100 μm; magnification, 40x. Semi-quantitative analysis illustrated that HBSP significantly attenuated total and interstitial apoptosis in both IR mice of genotypes, and further on *P^KO^*. **(E)** Representative images of F4/80 staining show macrophages in interstitial areas. Scale bar, 50 μm; magnification, 40x. Semi-quantitative analysis demonstrated that IR kidneys of *P^KO^* mice with HBSP treatment showed a lower level of macrophage infiltration than the *P^KO^* IR group and the WT control modified by HBSP. One-way ANOVA, Least significance difference (LSD) test. IR: n = 5-9; IR + HBSP: n = 4-6. Plots depict means ± SEM. *, *P*<0.05, **, *P*<0.01.

Previously, adult male C57BL/6 WT and *P^KO^* mice aged 8-12 week were subjected to bilateral renal ischaemia 30 min and reperfusion for 72 h, as well as sham surgery (WT sham: n = 4; *P^KO^* sham: n = 5; WT IR: n = 9; *P^KO^* IR: n = 8.) (12). Here, under equal standard of animal maintenance and anesthetic practice, the same IR surgical procedure was performed to additional *P^KO^* and their littermates WT mice with treatment of HBSP (**Figure 1A**).

HBSP (Science Peptide, Shanghai, China), dissolved in saline and warmed to 37°C, was given to animals at a total dose of 24 nmol/kg body weight through injecting at the onset of occlusion and 15 minutes after reperfusion (half/half) intraperitoneally. There were 6 or 5 mice in WT IR + HBSP group and *P^KO^* IR + HBSP group, respectively. Reperfusion was confirmed by the color of the renal surface changing from dark to patched blanching and then back to normal pink. All mice studies were performed in accordance with institutional guidelines approved by the United Kingdom Home Office. At 72 h of reperfusion, the animals were humanely killed and whole blood as well as bilateral kidneys were then obtained for further analysis.

### Biochemical detection

Serum Creatinine (SCr) and Blood urea nitrogen (BUN) levels were determined using a QuantiChromTM Creatinine Assay Kit and a Bioassay System urea assay kit (BioAssay System, Hayward, CA) according to the manufacturer’s instruction.

### Histological assessment

Four μm sections of paraffin-embedded kidney tissue were stained using hematoxylin & eosin. The score of tubulointerstitial damage (TID) in cortical areas was evaluated by two researchers blinded to the groups and treatment, using a previously described method (20). The sections were viewed at 400x magnification and 15 randomly selected cortical fields were scored for each kidney. The final TID for each animal was determined by dividing average scores from the left and right kidneys of each animal.

### Labeling apoptotic cells

*In situ* labeling of DNA strand breaks in apoptotic cells in paraffin-embedded kidney sections was done with the ApopTag^®^ Peroxidase kit (Merck, Watford, UK). The positively stained cells were revealed by AEC (3-amino-9-ethylcarbazole, bright red color) substrate (Vectorlabs, Newark, CA) and hematoxylin was used for counter staining. Apoptotic cells were separately examined in the tubular area, tubular lumen, and interstitial area of the renal cortex in 15 randomly selected fields at 400x magnification for each tissue. The final number of apoptotic cells in each animal was calculated by averaging the cell numbers from all fields of the left and right kidneys.

### Labeling macrophages

Interstitial infiltration of macrophages was analyzed by F4/80 staining. The paraffin-embedded kidney sections were dewaxed and treated with the EDTA Antigen Retrieval Solution (E673003, BBI, Shanghai, China) for 10 min in a high-pressure steam cooker. The sections were then processed to the QuickBlock blocking buffer (P0260, Beyotime, Shanghai, China) for 30 min at room temperature, followed by incubation with the primary rabbit–anti mouse F4/80 antibody (1:1000, 28463-1-AP, Proteintech, Chicago, USA) or normal rabbit IgG for negative control (2729S, CST, Danvers, USA) at 4°C overnight. The next day, the secondary antibody (PV-6000, ZSGB-BIO, Beijing, China) was applied to the sections for 20 min at 37°C. The 3,3′-diaminobenzidine (DAB, Beyotime) was used to reveal the antibody binding and then the sections were counterstained with hematoxylin. Positively stained cells were counted at 400× magnification for 15 randomly selected fields per section. The average of counts per field from both left and right kidneys in each animal was used for statistical analysis.

### Cell culture and treatment

TCMK-1, a mouse kidney epithelial cell line (ATCC, CCL-139), was maintained in the complete medium containing DMEM/F12 1:1 medium (Gibco, Paisley, UK), 10% fetal bovine serum (FBS, Sigma, Dorset, UK), 2 mM L-glutamine (Gibco), 100 U/ml penicillin G and 100 mg/ml streptomycin (Sigma), at 37°C in a 5% CO_2_ humidified atmosphere.

Cells at a density of 1×10^5^ cells/well were seeded into six-well plates and cultured in complete medium but without antibodies. The next day, the cells were changed culture medium to DMEM/F12 1:1 only and then transfected with siRNA (Thermo Fisher Scientific, Rockford, USA) targeting mouse properdin (PsiRNA, s71507), EPOR (EPORsiRNA, s65611) or negative control siRNA (NCsiRNA, 4390843, not targeting any known mammalian genes) at 20 nM with assistant of Lipofectamine™ RNAiMAX (Invitrogen, Carlsbad, USA).

Six hours after transfection, the cells were then changed culture medium to complete medium and stimulated with hydrogen peroxide (H_2_O_2_) at 100 μM to mimic oxidative stress during renal IR. At the same time, HBSP (Science Peptide) at 20 ng/ml was added to the cell culture medium for treatment. After another 18 hours, whole protein was extracted for detecting EPOR and EPOR/βcR by western blotting and co-immunoprecipitation. In addition, the percentage of early and later apoptotic cells was examined by Annexin V/PI staining (Roche, Mannheim, Germany) and determined by a flow cytometer (FACSCanto, BD, Bergen, USA). In each experiment, two replicates per group were used, while the individual experiment was repeated at least three times.

### Immunoblotting

The mice kidney and tubular cells were harvested and homogenized in RIPA lysis buffer (89900, Thermo Fisher Scientific). Twenty-five μg proteins were separated in SDS-PAGE gel and then transferred onto PVDF membrane (Merck, Watford, UK) at constant current of 300 mA for 1 h. The membrane was then blocked with 5% non-fat milk (Bio-Rad, Berkeley, USA) and incubated with Rabbit polyclonal primary antibody to EPOR (1:1,000, PAB18350, Abnova, Taiwan), PCNA (1:1,000, M0879, DAKO, Glostrup, Denmark) or β-actin (1:5,000, A5441, Sigma, Dorset, UK) overnight at 4°C. The secondary antibody (Goat–anti-Rabbit/Mouse, K4063, DAKO) was peroxidase-conjugated and incubated with the membrane for 2 h at room temperature. The membrane was developed using ECL substrate (Thermo Fisher Scientific) and a Molecular Imager ChemiDoc XRS+ system (Bio-Rad). Blots were semi-quantitatively analyzed by scanning volume density using Bio-Rad Image Lab Software 5.2.1 (Bio-Rad). Optical volume density values for target proteins were corrected by β-actin.

### Co-immunoprecipitation

The EPOR protein was immunoprecipitated from 200 μg tissue/cell homogenates through incubation with 1 μg of anti-EPOR antibody (PAB18350, Abnova) for overnight at 4 °C on a rotator. Then, 40 μl protein A sepharose beads (17-0469-01, GE healthcare, Pittsburgh, USA) was added to each sample and incubated for another 2 h at 4 °C on the rotator. Afterwards, the beads were collected by spinning at 500 g for 30 s and washed 3 times with RIPA buffer. The supernatant was discarded and 25 μl of 4×loading buffer (Bio-Rad) was added to each sample and boiled for 10 min at 100 °C on a heat block. The samples were separated by SDS-PAGE gels, transferred onto PVDF membranes and probed with anti-βcR antibody (sc-93281, Santa Cruz, Dallas, USA). The final detected βcR bands represent the level of EPOR/βcR heterodimer.

### HBSP localization in mice organs and tubular cells

For location tracking, HBSP was conjugated with iridium (Ir), which was produced by our collaborators in the Chemistry Department of University of Leicester.

*In vivo*, adult male C57BL/6 mice between 8-12 weeks, were subjected to bilateral renal ischaemia for 0 min (sham surgery) or 30 min followed by reperfusion for 17 h. Afterwards, HBSP-Ir conjugate (dissolved in sterile 0.9% saline) at 48 nmol/kg body weight was injected *via* the tail vein. Thirty minutes later, the mice were sacrificed and the kidneys, heart, liver and lungs were embedded in OCT and snap frozen in liquid nitrogen. Cryopreserved tissues from different organs were then cut into sections at 5 μm thickness using Leica freezing microtome (CM1950, Wetzlar, Germany), and mounted in Anti-Fade Fluorescence Mounting Medium (ab104135, abcam). The florescence signal (green) of Ir was excited at 405 nm and emission was collected between 500 +/- 20 nm using a confocal microscope (TCS SP8, Leica).

*In vitro*, TCMK-1 cells were seeded onto glass coverslips pre-coated with Poly-D-lysine (0.1 mg/ml; P1149, sigma). The density of seeding was 1.0×10^5^ cells/ml. After 24 h, the cells were stimulated with 200 μM of H_2_O_2_ for another 24 h. Then, HBSP, Ir or HBSP-Ir was added to the culture medium at a concentration of 50 μM. One hour later, the cells were labeled with Rhodamine Phalloidin (PHDR1, Cytoskeleton, Denver, USA) in order to visualize the cellular skeletal protein F-actin. The specimens were then mounted and observed using the confocal microscope (TCS SP8, Leica). Ir fluorescence was measured at the parameter same with the *in vivo* tracking. Rhodamine fluorescence was measured between 585 +/- 20 nm following excitation at 555 nm.

### Phagocytic assay

The phagocytic ability of TCMK-1 cells was evaluated *via* uptaking *E.coli* Bioparticles (FITC-labelled pHrodo *E.coli* Bioparticles^®^ Conjugate, P35366, Thermo Fisher Scientific) using flow cytometry. The fluorogenic dye conjugated to *E.coli* was pH sensitive that greatly increases in fluorescence when the surroundings becomes more acidic after phagocytosis occurs. TCMK-1 was seeded to 24-well plates at 1.0×10^5^ cells/ml and treated with HBSP at 20, 40 and 80 ng/ml next day for 24 h. *E.coli* Bioparticles (0.5 mg/ml, suspended in DMEM/F12 medium) was then added at 500 µl/well for 2 h. Afterwards, the cells were washed to remove non-phagocytosed *E.coli* Bioparticles and resuspended after tripsinizing. For each sample, a total of 10,000 gated cells were analysed for the fluorescent intensity of FITC on a flow cytometer (FACSCanto, BD). The average FITC intensity of total cells and positive cells were defined by a selected threshold; a percentage of positive cells was then analyzed.

### Statistical analysis

Data is expressed as mean ± standard error of the mean (SEM). One-way ANOVA and LSD (Least significance difference) tests were carried out by SPSS Statistics Standard V26.0 software (IBM, New York, USA) to assess differences between data means as appropriate. A *P* value of < 0.05 was considered statistically significant.

## Results

### HBSP protected 72-h IR kidneys in both genotype mice

The experimental design of a 72-h kidney IR injury model, illustrated in **Figure 1A**, used WT and *P^KO^* C57BL/6 mice with or without HBSP treatment. Our previous publication has included the data from sham and IR groups of both genotypes (12), of which values on the renal function, structural, apoptosis and PCNA have also been included in the **Table 1**. Here, we demonstrated the therapeutic effect of HBSP on kidneys from the two genotypes. At IR 72 h, the elevated SCr was significantly decreased by HBSP in both WT (1.2 0.2 vs. 0.6 0.1, *P* < 0.01) and *P^KO^* (1.8 0.3 vs. 0.8 0.2, *P* < 0.01) mice, with similar effects on BUN (**Figure 1B**). Structurally, the level of TID was increased by *P^KO^* in contrast to WT kidneys after IR injury, while HBSP treatment greatly reduced the TID score in both WT and *P^KO^* genotypes (**Figure 1C**). The number of apoptotic cells in the tubulointerstitial area of kidneys after IR was significantly reduced by HBSP, which was further decreased in *P^KO^* mice (WT IR + HBSP vs. *P^KO^* + IR + HBSP, 0.8 0.1 vs. 0.5 0.0, *P*<0.05, **Figure 1D**). Notably, the number of apoptotic cells was significantly lower in the interstitial area of IR kidneys from *P^KO^* mice than that in the WT control modified by HBSP. These results demonstrated that HBSP greatly ameliorated IR-induced kidney functional and structural injury at 72 h in both WT and *P^KO^* mice, and further decreased renal apoptotic level in *P^KO^* mice than in the WT control.

**Table 1:** Values of Sham vs. IR groups at IR 72 h.

Genotypes	Detections	Sham	IR	Statistics
WT	SCr	0.4 ± 0.1	1.2 ± 0.2	*P* < 0.05
	BUN	52.6 ± 2.9	82.3 ± 22.3	*P* < 0.05
	TID	1.2 ± 0.1	1.9 ± 0.2	*P* < 0.05
	Apoptosis	0.7 ± 0.1	2.9 ± 0.5	*P* < 0.05
	PCNA	1.0 ± 0.2	5.2 ± 1.1	*P* < 0.05
*P^KO^*	SCr	0.3 ± 0.1	1.8 ± 0.3	*P* < 0.01
	BUN	40.3 ± 2.3	246.4 ± 44.9	*P* < 0.01
	TID	1.4 ± 0.0	2.7 ± 0.3	*P* < 0.01
	Apoptosis	0.5 ± 0.2	4.5 ± 0.8	*P* < 0.01
	PCNA	2.9 ± 0.9	8.2 ± 1.5	*P* < 0.05

### HBSP further decreased macrophage infiltration in kidneys of *P^KO^* at IR 72 h

The immunostaining of F4/80 in the kidney revealed macrophage infiltration modified by IR and HBSP in both genotypes. There was no staining found in the negative control sections (results not shown). In the positively stained sections, the F4/80+ cells in the interstitial area were not significantly changed by HBSP in kidneys of WT mice, but were greatly reduced in *P^KO^* kidneys at IR 72 h (**Figure 1E**). Moreover, the *P^KO^* kidneys showed fewer positive cells than the WT control after treatment significantly (12.2 0.8 vs. 18.4 1.9, *P*<0.05). Thus, HBSP treatment decreased the number of interstitial F4/80+ cells in IR kidneys of *P^KO^* mice only, which also showed a considerable reduction of F4/80+ cells than in the WT control after treatment.

### EPOR, EPOR/βcR and PCNA expression were regulated by IR, *P^KO^* and HBSP

**Figure 2:**
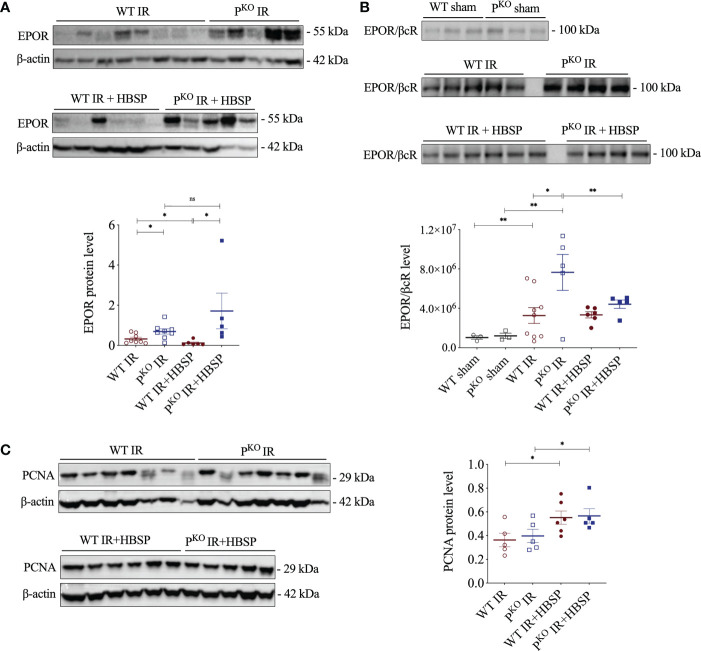
At 72 h, HBSP significantly reduced the high of EPOR proteins in WT IR mice and the heterodimer EPOR/βcR in *P^KO^* IR mice. **(A)** HBSP decreased the high levels of EPOR in WT IR mice but not in *P^KO^* IR mice by western blotting corrected with β-actin. **(B)** Co-immunoprecipitation demonstrated that the expression of EPOR/βcR protein complex was IR significantly increased in both WT and *P^KO^* kidneys after IR, but reduced by HBSP only in *P^KO^* kidneys. **(C)** HBSP increased the level of PCNA in IR mice of both WT and *P^KO^* mice by western blotting corrected with β-actin. One-way ANOVA, LSD test. Sham: n = 3; IR: n = 5-8; IR + HBSP: n = 4-6. Plots depict means ± SEM. *, *P*<0.05, **, *P*<0.01, ns, no significance.

EPOR was previously found upregulated by IR and enhanced by *P^KO^* at IR 72 h (12), thus, the expressional change of EPOR, especially EPOR/βcR, in IR kidneys regulated by HBSP was further explored. The highly expressed EPOR in IR kidneys was greatly downregulated by HBSP in WT mice, but not in *P^KO^* mice (**Figure 2A**). In fact, the level of EPOR in *P^KO^* IR kidneys was still significantly higher than that in the WT control (1.7 0.8 vs. 0.1 0.0, *P*<0.05) after HBSP treatment. In addition, the expression of the heterodimer EPOR/βcR was greatly increased by IR injury in both genotypes and furthered by *P^KO^* (7.6×10^6^ 1.8×10^6^ vs. 3.2×10^6^ 8.0×10^5^, *P*<0.05, **Figure 2B**). HBSP treatment significantly decreased the level of EPOR/βcR in the kidneys of *P^KO^* mice, but not in WT mice after IR. The expression of PCNA protein was significantly upregulated by HBSP in both genotypes (**Figure 2C**). HBSP increased the PCNA in IR kidneys of both genotypes but modified the tissue protective receptors EPOR and EPOR/βcR differentially in WT and *P^KO^* mice kidneys.

### HBSP located in tubular epithelial cells post IR and IR-related injury

**Figure 3:**
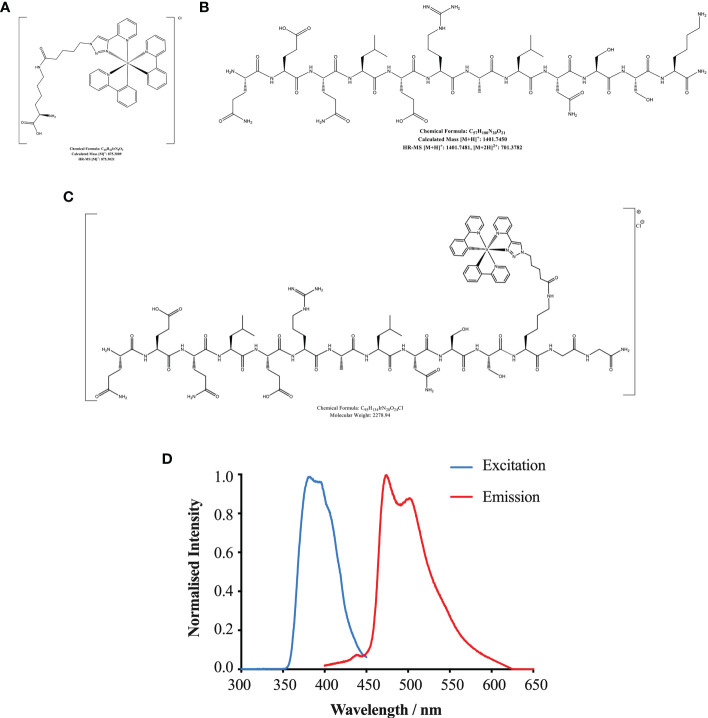
Chemical formulas of Iridium **(A)**, HBSP **(B)** and iridium (Ir)-labelled HBSP **(**HBSP-Ir, **C)**. **(D)** Normalized excitation and emission profile of complex HBSP-Ir.

**Figure 4:**
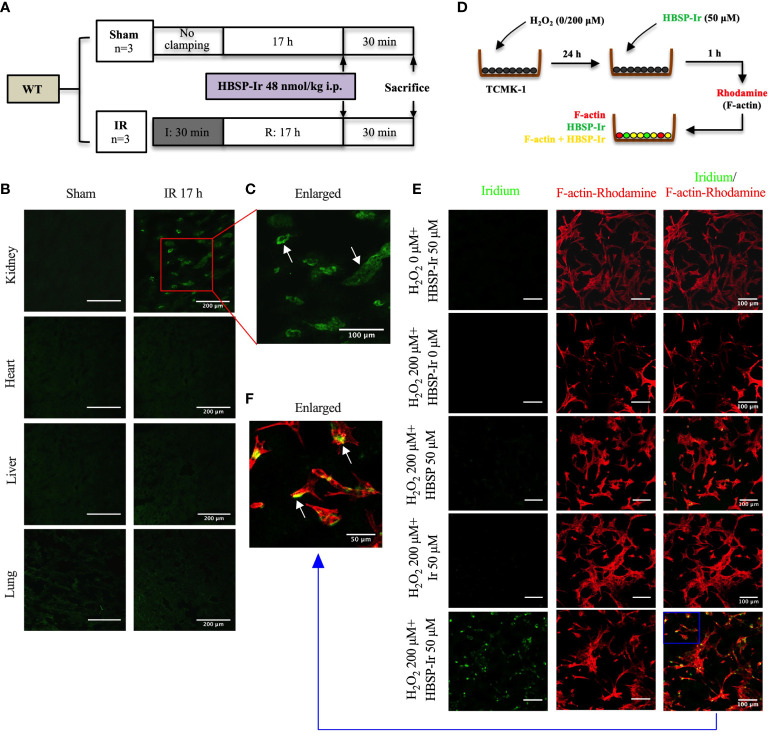
HBSP anchors on tubular epithelial cells under IR or IR-related stimulation both *in vivo and in vitro*. **(A)** The experimental design of mouse kidney IR models treated with HBSP-Ir at 17 h. **(B, C)** Representative micrographs from the kidney, heart, liver and lung of sham or IR mice show HBSP-Ir mainly locates on renal TECs, with some weak signals in lung alveolus. The boxed area is enlarged. Scale bar, 200 μm/100 μm (the enlarged image); magnification: 40×. **(D)** The *in vitro* study design of HBSP-Ir localization in tubular epithelial cell (TCMK-1) treated with H_2_O_2_. **(E, F)** TCMK-1 cells treated with both H_2_O_2_ and HBSP-Ir showed significant co-localization of HBSP-Ir (green) and F-actin (Rhodamine, red). Scale bar, 100 μm/50 μm (the enlarged image); magnification: 40×.

The structure and chemical formulas of Ir, HBSP and HBSP-Ir are shown in **Figures 3A–C** respectively. The excitation and emission wavelengths of Ir were 300-450 nm and 400-600 nm (shown as green signals), respectively (**Figure 3D**). HBSP-Ir was used to track the localization of HBSP in major organs and cells in the context of renal IR injury (**Figure 4A**). As shown in **Figure 4B**, there was no recognizable green signal (seen as Ir) in the kidney of sham animals, nor in the heart and liver (left panel). However, at IR 17 h, the green signal was greatly distributed in the kidney and mainly localized at tubules (right panel). High magnification pictures demonstrated that the green signal was localized on the apical surface of tubules (**Figure 4C**). Green signals were also found in the lungs of both sham animals and IR animals, but much weaker than that in IR kidneys.

*In vitro*, HBSP, Ir or HBSP-Ir was used to treat H_2_O_2_-stimulated TCMK-1 cells for tracking HBSP-Ir in contrast to the Rhodamine-labelled F-actin (Red, **Figure 4D**). TCMK-1 cells treated with both HBSP-Ir and H_2_O_2_ demonstrated marked green signals (**Figure 4E**), which was around or overlapped with F-actin (Orange, **Figure 4F**). However, cells treated with H_2_O_2_ only, H_2_O_2_ + HBSP or H_2_O_2_ + Ir, and even HBSP-Ir only, did not show visible green signals. Both *in vivo* and *in vitro* tracking of HBSP-Ir demonstrated that renal tubular epithelial cells are the main target during IR and related injury.

### Properdin knockdown increased EPOR expression and affected apoptosis in tubules

**Figure 5:**
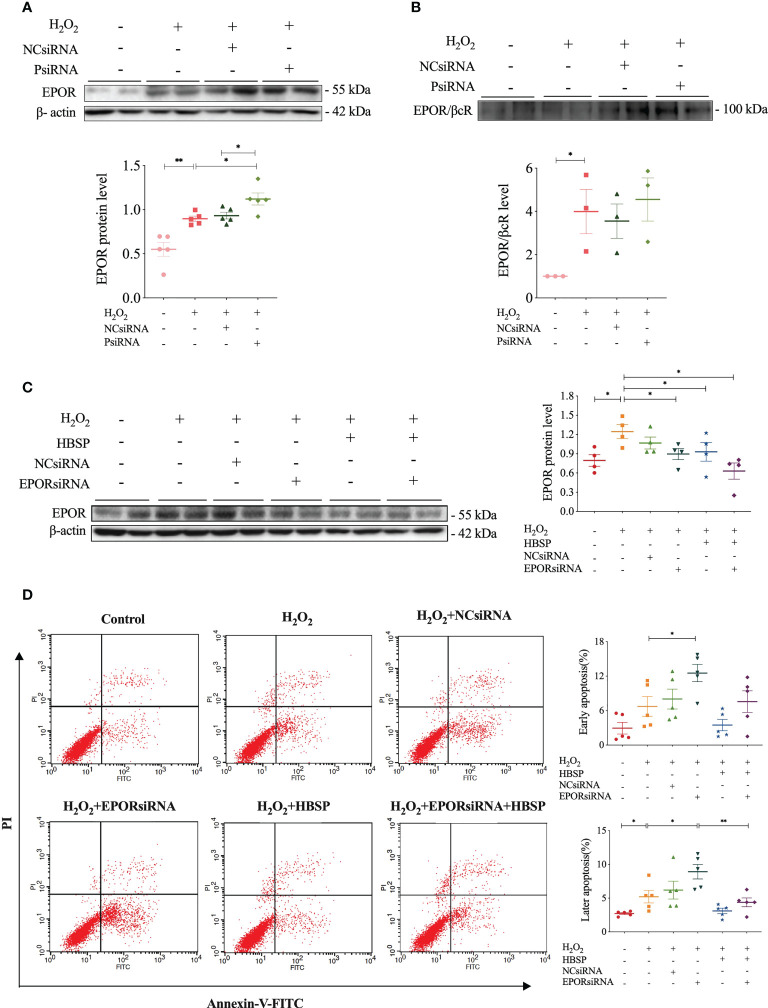
Properdin silencing increased EPOR expression that mediated cell apoptosis revealed in kidney tubular epithelial cells. **(A)** Small interfering RNA targeting mouse properdin (PsiRNA) significantly increased the EPOR level in TCMK-1 cells stimulated with H_2_O_2_ by western blotting, corrected with β-actin. Five independent experiments with 2 replicates each time. **(B)** Co-immunoprecipitation shows that H_2_O_2_ greatly increased EPOR/βcR protein complex in TCMK-1, but remained a similar level under PsiRNA interfering. Three independent experiments with 2 replicates each time. **(C)** Western blot shows that the level of EPOR protein in TCMK-1 cells was decreased by small interfering RNA targeting mouse EPOR (EPORsiRNA), HBSP or EPORsiRNA + HBSP significantly under stimulation with H_2_O_2_. Four independent experiments with 2 replicates each time. **(D)** Flow cytometry data demonstrates that H_2_O_2_ treatment significantly increases % late apoptosis (upper right quadrant), furthered by EPORsiRNA, but decreased by HBSP. EPORsiRNA also increased % early apoptosis (lower right quadrant) under H_2_O_2_ treatment. Five independent experiments with 2 replicates each time. One-way ANOVA, LSD test. Plots depict means ± SEM. *, *P*<0.05, **, *P*<0.01.

The relationship between properdin and EPOR or EPOR/βcR expression, as well as the association between EPOR expression and tubular apoptosis, was further explored by using relevant siRNA *in vitro*. Western blotting results showed that H_2_O_2_ stimulation significantly increased the expression of EPOR and EPOR/βcR in TCMK-1 cells (**Figures 5A, B**). Moreover, PsiRNA further upregulated the EPOR level, but not EPOR/βcR, in comparison with NCsiRNA (**Figure 5A**). The knockdown efficacy of PsiRNA used here was evaluated previously (12). Using EPORsiRNA or HBSP, the EPOR expression was greatly reduced in TCMK-1 cells after H_2_O_2_ treatment (**Figure 5C**). However, Flow cytometry analysis showed that EPORsiRNA significantly increased the level of early and late apoptosis in H_2_O_2_-stimulated TCMK-1 cells, of which the percentage of raised late apoptosis was attenuated by HBSP (**Figure 5D**). These results showed that knocking down of properdin further elevated the EPOR expression in TCMK-1 cells upon H_2_O_2_ stimulation, and the maintenance of the high EPOR expression controls the apoptotic level in TCMK-1 cells.

### HBSP enhanced the phagocytic function of kidney tubular epithelia

**Figure 6:**
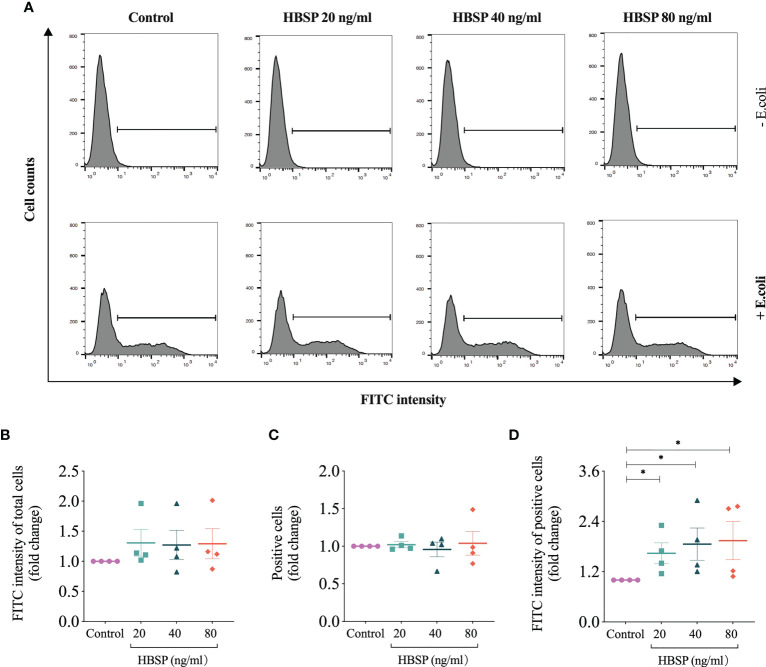
HBSP treatment enhances the phagocytic function of tubular epithelial cell *in vitro*. **(A)** Flow cytometry analyses TCMK-1 cells uptaking FITC-labeled *E.coli*. Cells under the threshold line were seen as positive. **(B-D)** The fold change of the average intensity of FITC fluorescence among total analyzed cells, positive cell counts and the average intensity of FITC fluorescence among positive cells are shown against the corresponding group without *E.coli* treatment. Four independent experiments with 2 replicates each time. One-way ANOVA, LSD test. Plots depict means ± SEM.*, *P*<0.05.

The role of HBSP on the phagocytic function of kidney tubular epithelia was assessed by the uptake of FITC fluorescent-labeled *E.coli* and detected by flow cytometry (**Figure 6A**). In contrast to the control cells, there were no significant differences in TCMK-1 cells treated with gradient doses of HBSP, demonstrated by fold changes in the average fluorescent intensity of total cells and positive cells (**Figures 6B, C**). However, the fold change in average fluorescent intensity of positive cells was significantly increased by HBSP dose-dependently compared to the control (**Figure 6D**). Thus, HBSP treatment enhanced the phagocytic function of TCMK-1 cells.

## Discussion

It has been widely reported, including our previous studies that HBSP, a non-erythropoietic peptide derived from EPO, remarkably ameliorated IR-induced kidney damage with notable improvement in cell death (7, 21–23). However, the mechanism of HBSP renoprotection has not been fully defined, in particular its effect on phagocytosis associated with properdin. In the present study, HBSP significantly improved IR kidney injury in *P^KO^* mice in terms of reducing cell apoptosis and macrophage infiltration, while *P^KO^* IR kidneys had more severe damage than WT controls. Properdin was shown to be an essential opsonizing molecule of phagocytic process, and also involved in the phagocytic function of renal TECs (12, 24). The protective effect of HBSP against IR injury in *P^KO^* kidneys might attribute to the higher expression of EPOR/βcR in TECs, by which HBSP might boost the phagocytic function of TECs as a compensation mechanism for the impact of properdin deficiency on phagocytosis.

HBSP, a specific ligand of the tissue protective receptor EPOR/βcR, reversed renal IR-induced functional and structural damage and promoted proliferation, not only in the kidneys of WT mice, but also more effective in severe damaged kidneys of properdin deficient mice. We demonstrated that the expression of EPOR/βcR in kidneys was greatly increased by the absence of properdin after IR injury, which was also reliant upon the level of elevated EPOR. The upregulated expression of these receptors would facilitate renoprotection of HBSP treatment in terms of compensating the absence of properdin opsonized phagocytosis. In addition, it was worth to note that HBSP treatment led to fewer interstitial apoptotic cells as well as F4/80+ macrophages in *P^KO^* mouse kidneys compared with the WT control. Apoptotic cells in interstitial areas are often regarded as remnants of dead infiltrated inflammatory cells after they finished the mission of clearing acute inflammation. Thus, the fewer apoptosis in the interstitial area would result from the less macrophage infiltration in the *P^KO^* IR kidneys. The location of heterodimer EPOR/βcR was found in the tubules of WT and *P^KO^* mouse kidneys after IR (7, 12). The highly expressed EPOR/βcR in *P^KO^* mouse kidneys could contribute to the better preservation of renal parenchymal cells including TECs, thus reduce tissue injury and macrophage infiltration. In addition, it has been reported that HBSP could reduce the secretion of pro-inflammatory cytokines from macrophages, as well as the transformation of macrophages towards pro-inflammatory M1 genotype, but promote their survival by upregulating the expression of caspase activation inhibitors including survivin and Bag1 (10, 25). These evidences indicated the direct effect of HBSP on inflammation including not only infiltration, but also releasing cytokines and genotype transformation. Thus, HBSP *via* the highly expressed EPOR/βcR, in particular in *P^KO^* mouse kidneys, modulated the biological function and fate of both inflammatory cells and tubular cells including the proportion of apoptotic cells and F4/80+ macrophages in interstitial areas, subsequently balanced immune responses in the acute stage of AKI.

The location and type of EPOR/βcR expressing cells were further verified in kidneys and other organs by conjugating HBSP with iridium that emits green florescence upon excitation. Tracking HBSP-Ir using confocal microscope showed that strong green signals were mainly in the apical surface of tubular lumen in the kidneys after IR for 17 h, but not in the sham controls. Weak signals were seen in the heart and liver, as well as both sham and IR groups. Although the lung showed weaker signals than in the kidney, it had stronger signal than in the heart and liver in the IR group would attribute to the auto-fluorescent from the lung as shown in the sham group. *In vitro*, TCMK-1 cells were further used to confirm the binding of HBSP-Ir with EPOR/βcR upon IR-related oxidative stress at 24 h. Intriguingly, HBSP treatment modulated EPOR and EPOR/βcR expression in both genotype mouse kidneys, but in a different manner. The level of EPOR protein was significantly decreased by HBSP in WT mouse kidneys, but still remained high in *P^KO^* mouse kidneys after IR. However, EPOR/βcR in the IR kidneys was significantly reduced by HBSP in the *P^KO^* mice only. It was reported that the high level of EPOR in the kidney leads to renal tubulointerstitial fibrosis 2 weeks after IR (26). Therapeutic EPO stimulated profibrotic factors and promoted fibrosis and myofibroblast proliferation 4 weeks post IR, which might be due to its higher affinity to (EPOR)_2_ than EPOR/βcR (27). Properdin deficiency further upregulated the expression of EPOR/βcR in IR kidneys associated with more severe injury, but also initiated stronger repair and better responses to the treatment of its ligand HBSP. However, the constant highly expressed EPOR in the kidney of *P^KO^* mice before and after HBSP treatment might indicate potential negative impact of EPOR towards late stage of renal IR (26).

To further explore the association between properdin and EPO receptors, as well as their biological significance in IR kidneys, siRNA target both genes were applied in cultured TCMK-1 cells. Silencing properdin in TCMK-1 cells significantly upregulated the expression of EPOR protein under oxidative stress, indicating a complementary regulation between properdin and EPOR expression or an intrinsic balance *via* EPOR against properdin deficiency-mediated damage (12). However, in the above cell model, the expression of EPOR/βcR was not significantly upregulated by silencing properdin, which may be resulted from the limited observation period of 24 h *in vitro* compared to 72 h *in vivo*. We also demonstrated that the basic level of EPOR expression is essential for maintaining cell survival as silencing EPOR increased the number of apoptotic TCMK-1 cells. It has been found that EPO/EPOR signaling could enhance the uptake of apoptotic cells by macrophages and improve immune tolerance (11). It has also been reported that macrophage EPO signaling is temporally induced during infections, which increases engulfing bacteria, promotes infection resolution, and lowers antibiotic requirements (28). Here, it is the first time to show that HBSP greatly increased the phagocytic function of TCMK-1 cells assessed by uptaking *E.coli* Bioparticles. This implies that the protective role of EPOR/βcR signaling in TECs is associated with the phagocytic efficacy of TECs. As a result, renal parenchymal cells were then preserved and tissue injury was reduced by the timely clearance of dead cells. Thereby, the further increased EPOR/βcR in *P^KO^* mouse kidneys could subsequently promote the phagocytic function of TECs to limit the damage level of IR kidneys caused by properdin deficiency and compromised phagocytosis, and also enhanced the therapeutic effect of HBSP.

There are limitations in this study. The relationship between properdin, EPOR and EPOR/βcR could be further explored *in vivo* and *in vitro* in a time-course model by modifying gene expression during renal IR-related injury. The significance of high level of EPOR in IR kidneys of *P^KO^* mice with or without HBSP treatment would be further studied, as well as the long-term biological role of EPOR and EPOR/βcR would be differentiated. In addition, the phagocytic function demonstrated by HBSP would be further verified in TECs subjected to IR-related injury.

## Conclusion

HBSP protected kidneys against IR not only in WT mice, but also with enhanced renoprotection in *P^KO^* mice. The elevated EPOR and EPOR/βcR by *P^KO^* in IR kidneys might initiate repair, sensitizing them to HBSP treatment, subsequently leading to less cell apoptosis and inflammation, and kidney restoration. The relationship between HBSP, properdin, EPOR and EPOR/βcR and its related biological functions are worthy of further study.

## Data availability statement

The raw data supporting the conclusions of this article will be made available by the authors, without undue reservation.

## Ethics statement

The animal study was reviewed and approved by United Kingdom Home Office.

## Author contributions

BY and YW designed the study. YW and ZZ carried out animal experiments. LH, WS, FC conducted analysis of animal specimens. YL and CH performed cell experiments. JB and ML synthesized HBSP-Ir. YW, LH and WS analyzed the data and made the figures. YW, BY and NB drafted and revised the paper. All authors contributed to the article and approved the submitted version.
