# Corrigendum: HBSP improves kidney ischemia-reperfusion injury and promotes repair in properdin deficient mice via enhancing phagocytosis of tubular epithelial cells

**DOI:** 10.3389/fimmu.2023.1242436

**Published:** 2023-06-29

**Authors:** Yuanyuan Wu, Lili Huang, Wenli Sai, Fei Chen, Yu Liu, Cheng Han, Joanna M. Barker, Zinah D. Zwaini, Mark P. Lowe, Nigel J. Brunskill, Bin Yang

**Affiliations:** ^1^ Department of Pathology, Medical School of Nantong University, Nantong, China; ^2^ Department of Cardiovascular Sciences, College of Life Sciences, University of Leicester, University Hospitals of Leicester NHS Trust, Leicester, United Kingdom; ^3^ Nantong-Leicester Joint Institute of Kidney Science, Nephrology, Affiliated Hospital of Nantong University, Nantong, China; ^4^ Research Center of Clinical Medicine, Affiliated Hospital of Nantong University, Nantong, China; ^5^ School of Chemistry, University of Leicester, Leicester, United Kingdom; ^6^ Department of Respiratory Sciences, College of Life Sciences, University of Leicester, Leicester, United Kingdom

**Keywords:** HBSP, innate repair receptor, ischaemia-reperfusion injury, phagocytosis, properdin, repair, tubular epithelial cells

In the published article, an author name was incorrectly written as Weili Sai. The correct spelling is Wenli Sai.

The authors apologize for this error and state that this does not change the scientific conclusions of the article in any way. The original article has been updated

